# Literature-based occurrences data of marine species in Venezuela

**DOI:** 10.3897/BDJ.11.e98213

**Published:** 2023-02-03

**Authors:** Ana Carolina Peralta Brichtova, Joxmer Scott-Frías, Carlos Carmona-Suarez, Carmen Teresa Rodriguez, Jeannette Perez-Benítez, Adriana Lopez-Ordaz, Brightdoom Marquez-Rojas, Carlos Lira, Santiago Gómez Acevedo, Yusneyi Carballo Barrera, Bladimir Rodríguez, Francoise Cavada-Blanco, José Ramón Delgado, Eduardo Klein

**Affiliations:** 1 INTECMAR, Caribbean OBIS, Fundación Caribe Sur, Caracas, Venezuela INTECMAR, Caribbean OBIS, Fundación Caribe Sur Caracas Venezuela; 2 Línea de Investigación del Plancton, Instituto de Zoología y Ecología Tropical, Universidad Central de Venezuela, Caracas, Venezuela Línea de Investigación del Plancton, Instituto de Zoología y Ecología Tropical, Universidad Central de Venezuela Caracas Venezuela; 3 IVIC, Caracas, Venezuela IVIC Caracas Venezuela; 4 BioMaC, Universidad de Carabobo, Valencia, Venezuela BioMaC, Universidad de Carabobo Valencia Venezuela; 5 Instituto de Zoología y Ecología Tropical, Universidad Central de Venezuela, Caribbean OBIS, Caracas, Venezuela Instituto de Zoología y Ecología Tropical, Universidad Central de Venezuela, Caribbean OBIS Caracas Venezuela; 6 Universidad Simón Bolívar - INTECMAR, Caracas, Venezuela Universidad Simón Bolívar - INTECMAR Caracas Venezuela; 7 Universidad de Oriente - IOV, Cumana, Venezuela Universidad de Oriente - IOV Cumana Venezuela; 8 Universidad de Oriente - ECAM, Boca del Río, Venezuela Universidad de Oriente - ECAM Boca del Río Venezuela; 9 Instituto de Biología Experimental, Universidad Central de Venezuela, Caracas, Venezuela Instituto de Biología Experimental, Universidad Central de Venezuela Caracas Venezuela; 10 Centro de Enseñanza Asistida por Computador (CENEAC), Escuela de Computación, Universidad Central de Venezuela, Caracas, Venezuela Centro de Enseñanza Asistida por Computador (CENEAC), Escuela de Computación, Universidad Central de Venezuela Caracas Venezuela; 11 Fundación Museo del Mar, Boca del Río, Venezuela Fundación Museo del Mar Boca del Río Venezuela; 12 Fundación Caribe Sur, Caracas, Venezuela Fundación Caribe Sur Caracas Venezuela; 13 Universidad Simon Bolivar, Caribbean OBIS, Caracas, Venezuela Universidad Simon Bolivar, Caribbean OBIS Caracas Venezuela

**Keywords:** occurrence data, marine species, biodiversity, data digitisation, Animalia, Chromista, Bacteria, Plantae, Fungi, Protozoa, OBIS, GBIF, Venezuela, southern Caribbean.

## Abstract

**Background:**

Venezuela has suffered a severe academic and research management crisis and funding opportunities for marine research and data management have been practically absent. This has worsened over the past five years and, as a result, libraries and other institutional spaces have been repeatedly vandalised, with hundreds of records, specimens and historical data stolen, destroyed or burned. To avoid the loss of irreplaceable data on Venezuelan biodiversity, an initiative was promoted, aimed at digitising information to create a rich dataset of biodiversity records, with emphasis on marine protected areas for the country, as well as to fill gaps in the distribution and status of marine biodiversity in Venezuela. Nighteen (19) institutions in the country focusing on marine science have consistently produced a wealth of information about Venezuela’s marine biodiversity in the form of specimen collections, unpublished sampled data and research theses through the work of hundreds of researchers and students. An inventory of available data sources at these national institutions was conducted under the National Biodiversity Data Mobilization Grant and the Biodiversity Information for Development Program, together with the Global Biodiversity Information Facility (GBIF) support. All recovered and processed datasets were published in the Ocean Biodiversity Information System (OBIS) and the Global Biodiversity Information Facility (GBIF) repositories.

**New information:**

This occurrences data collection represents a major contribution to the marine biodiversity inventory in Venezuela. It is based on numerous published papers, reports, books and checklists provided by experts, covering a broad taxonomic collection from which we obtained species occurrences (present and absent), organised into 59 datasets containing 40,881 records. This represents a 28.49% contribution to the records of the Venezuelan marine biodiversity reported to the OBIS (143,513 records in the OBIS until November 2022). The extracted data showed 3,041 marine species, with representatives of each of the six kingdoms: Animalia, Chromista, Bacteria, Plantae, Fungi and Protozoa. The datasets provide information on occurrence since 1822, extending the temporal coverage of the species occurrence inventory for Venezuela, which was established in 1879 before this project. The number of records for Venezuela increased by 41.3% compared with the data available before the project. Most of the occurrences (63.47%) were registered in Marine Protected Areas. Data collection included records of non-native species, descriptions of new species and species listed under different IUCN categories.

## Introduction

Venezuela is amongst the top ten countries with the greatest biodiversity in the world (Aguilera et al. 2003, Grande 2018). However, due to the enormous impact of human activities, such as tourism, overexploitation of marine resources, physical alteration and pollution, marine environments are at great risk and their biodiversity is highly threatened (Miloslavich et al. 2003). Coastal area management involves assessing changes in the distribution and abundance of coastal and marine species. However, the Venezuelan Integrated Plan for Coastal Management (Plan de Ordenamiento y Gestión Integral de Zonas Costeras) reveals a lack of information related to biodiversity attributes and indicators, which forms the basis for projecting risks and identifying actions to reduce coastal vulnerability (Minamb 2013, Peralta Brichtova 2021). On the other hand, Venezuela is suffering a severe academic and research management crisis and funding opportunities for marine research and data management have been practically absent (Requena 2003, Requena 2012, Bull and Rosales 2020, Van Roekel and De Theije 2020, Garcia Zea 2020, Requena 2021). This has worsened over the past five years and, as a result, libraries and other institutional spaces have been repeatedly vandalised with hundreds of records, specimens and historical data stolen, destroyed or burned. To preserve the information that will serve assessments, planning and management, an initiative for mobilising marine data was promoted by Fundación Caribe Sur. Through the “Rescuing the knowledge base of Venezuela’s marine biodiversity” project supported by the Global Biodiversity Information Facility (GBIF) and funded by the European Union via the Biodiversity Information for Development Programme-BID, the project managed to identify and digitise the Venezuelan marine biodiversity data found in articles and grey literature stored in many national academic institutions. This article summarises the rescued dataset collections derived from this project, which are hosted in the Ocean Biodiversity Information System (OBIS) and GBIF to date. The resulting data collection is composed of 59 datasets (occurrence and sampling events) with 40,881 records of marine organisms from a broad range of taxonomic categories registered within the Venezuelan maritime area, including some of its islands (Table 1).

## Project description

### Title

Rescuing the knowledge base of Venezuela’s marine biodiversity

### Personnel

Ana Carolina Peralta Brichtova, Joxmer Scott-Frías, Carlos Carmona-Suárez, Carmen Rodriguez, Jeannette Perez, Adriana Lopez Ordaz, Brightdoom Marquez, Carlos Lira, Santiago Gómez Acevedo, Yusneyi Carballo Barrera, Bladimir Rodríguez, Francoise Cavada-Blanco, José Ramón Delgado and Eduardo Klein Salas

### Design description

Fundación Caribe Sur, supported by the Global Biodiversity Information Facility-GBIF, carried out the project “Rescuing the knowledge base of Venezuela’s marine biodiversity”. This Project convened researchers affiliated to seven national academic institutions and two NGOs (Universidad Simón Bolívar, Universidad Central de Venezuela, Universidad de Carabobo, Universidad de Oriente, Instituto Venezolano de Investigaciones Científicas, Universidad Nacional Experimental Francisco de Miranda, Universidad del Zulia, Fundación Museo del Mar - Museo Marino de Margarita, Fundación Caribe Sur) to safeguard the largest amount of information on marine biodiversity that has been produced in the country. The project participants rescued data on marine biodiversity from most Venezuelan marine areas by digitising and mobilising information on marine biodiversity found in each of the national institutions mentioned above. Consequently, the project integrated national researchers into the community of contributors and users of georeferenced biodiversity data of Venezuelan marine environments.

### Funding

The resources to undertake this project have been received from the European Union and GBIF under the National Biodiversity Data Mobilisation Grant and the Biodiversity Information for Development Programme - BID implemented in the Caribbean region (led by GBIF), under Proyecto GBIF-Caribe Sur / ID: BID-CA2020-025-NAC "Rescate de la Data sobre Biodiversidad Marina en Venezuela"

## Sampling methods

### Sampling description

Data collection, curation and digitisation were performed by a team of 14 researchers affiliated to the most important universities, scientific research centres and NGOs that deal with marine science and marine management in Venezuela. The work contains literature-based sampling information on marine organism occurrences collected from institution libraries from which theses, research project reports and journal publications were reviewed (Table 2) to obtain data on the taxonomic groups, location of occurrence, collection dates, measurements of habitat features (such as physical and chemical parameters of the environment), biotic measurements (e.g. body size, abundance and biomass) and details regarding the nature of the sampling or observation methods, equipment and sampling effort.

### Quality control

All data were structured into the Darwin Core Biodiversity Standard (Wieczorek et al. 2012), adopting the OBIS Darwin Core template and OBIS-ENV-DATA structure (OBIS 2022). The datasets were created according to the data source, taxonomic groups, home institution and professional expertise of the scientists involved in data digitisation.

## Geographic coverage

### Description

**Figure 1.**
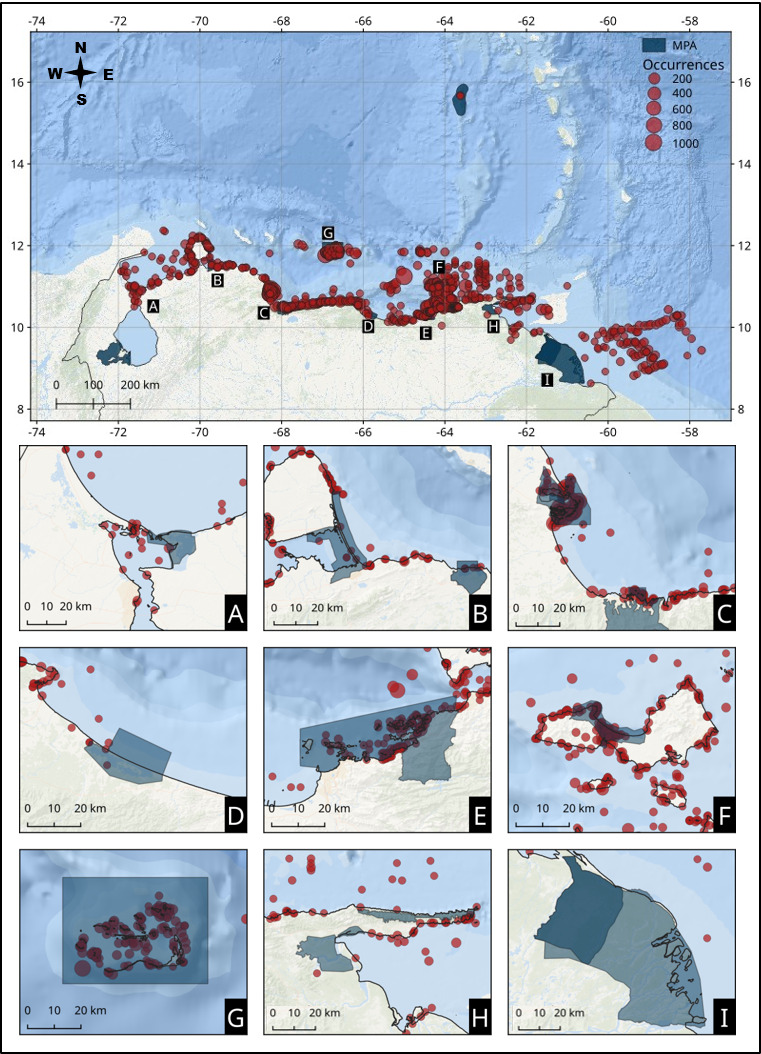
Location and aggregation of occurrences reported in this work for the Venezuelan coast and its islands. Dark blue regions represent MPAs: **A** Ciénaga de los Olivitos National Park; **B** Médanos de Coro National Park, Laguna Boca de Caño Wildlife Refuge and Hueque-Sauca Wildlife Reserve; **C** Morrocoy and San Esteban National Parks, Cuare Wildlife Refuge; **D** Laguna de Tacarigua National Park; **E** Mochima National Park; **F** Laguna de La Restinga National Park; **G** Archipiélago de Los Roques National Park; **H** Península de Paria and Turuépano National Parks; **I** Mariusa National Park and Delta del Orinoco Biosphere Reserve.

The data coverage was extracted directly from the literature and checked for any misreported georeferences, covered the entire Venezuelan mainland coast and some of its islands, including diverse marine coastal habitats, such as coral reefs, mangroves, rocky shores, sandy beaches, seagrass beds, coastal lagoons, sandy bottoms, oceanic water column and sea floor. Most occurrences (63.45%) were registered within marine protected areas, including seven national parks (Morrocoy, La Restinga, Archipiélago de Los Roques, San Esteban, Mochima, Médanos de Coro and Península de Paria) and four wildlife refuges (Cuare, Boca de Caño, Hueque-Sauca and Isla de Aves) (Table 3, Fig. 1). However, some MPAs (Laguna de Tacarigua, Turuépano, and Mariusa) still show important gaps in their biodiversity records. This project recorded new occurrences from areas that traditionally lacked biodiversity information, such as the Orinoco Delta and Atlantic Front, Paria Peninsula and coastal areas of western Venezuela states (Falcón and Zulia).

### Coordinates

8.612 and 15.676 Latitude; -71.939 and -57.705 Longitude.

## Taxonomic coverage

### Description

The taxonomic structure of the Venezuelan marine biodiversity collection at the time of publication represents a total of 30 Phyla, belonging to the kingdoms Animalia (17), Chromista (6), Plantae (4), Bacteria (1), Fungi (1) and Protozoa (1) (Table 4). The total number of records identified at the species level was 34,615, representing 84.67% of all the records. The remaining 15% of the records were identified at the family and genus levels.

A total of 3,041 species are reported. Most records belong to the phylum Arthropoda (10,642 records, 375 spp.) and phylum Rhodophyta (6,306 records, 322 spp.), while the least represented phylum were Bacteroidetes, Brachiopoda, Cryptophyta and Planctomycetes, with one single record each.

The data included records of non-native species, new species descriptions (*Spongicolaliosomatus*, *Haplophragmoidesvenezuelanus* and *Neopateorislopsischichirivensis*) and 78 species listed under different Threatened and Near Threatened IUCN categories (IUCN 2021): five species Critically Endangered (CR), nine Endangered (EN), 43 Vulnerable (VU) and 21 Near Threatened (NT) (Table 5).

## Temporal coverage

**Data range:** 1822-1-01 – 2022-4-13.

### Notes

**Figure 2.**
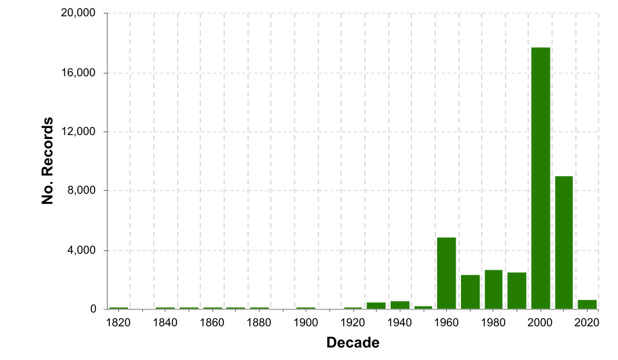
Historical series for Venezuelan marine species occurrences.

The data records extracted from literature have at least a year of collection. They include records from 1822 to 2022 (Fig. 2). Most occurrences were registered in the 1960s onwards, with the largest number of documented records in the 2000 decade.

## Usage licence

### Usage licence

Other

### IP rights notes

Creative Commons Attribution Non Commercial (CC-BY-NC) 4.0 License

## Data resources

### Data package title

Rescuing the knowledge base of Venezuela’s marine biodiversity

### Resource link

https://www.gbif.org/project/BID-CA2020-025-NAC/rescuing-the-knowledge-base-of-venezuelas-marine-biodiversity#datasets

### Number of data sets

1

### Data set 1.

#### Data set name

Events and occurrences of marine species data digitisation in Venezuela

#### Data format

DwC & GBIF API terms; UTF-8 character encoding

#### Download URL

https://doi.org/10.15468/dl.4tu8q5

#### Data format version

Darwin Core Archive 1.6

#### Description

The database provides information on observations since 1822, including a broad taxonomic group of marine organisms compiled from 59 datasets (Table 1) with a total of 40,881 records. Most datasets are structured using Event Core Schema with Occurrences and Extended Measurements or Facts (eMOF) extensions; therefore, they contain not only georeferenced occurrence records, but also sampling protocols and environmental and biotic measurements.

**Data set 1.** 

Column label	Column description
identifier	A related resource that is referenced, cited or otherwise pointed to by the described resource.
licence	A legal document giving official permission to do something with the resource.
basisOfRecord	The specific nature of the data record.
occurrenceID	An identifier for the Occurrence (as opposed to a particular digital record of the occurrence). In the absence of a persistent global unique identifier, construct one from a combination of identifiers in the record that will most closely make the occurrenceID globally unique.
occurrenceStatus	A statement about the presence or absence of a Taxon at a Location.
eventDate	The date-time or interval during which an Event occurred. For occurrences, this is the date-time when the event was recorded. Not suitable for a time in a geological context.
year	The four-digit year in which the Event occurred, according to the Common Era Calendar.
scientificNameID	An identifier for the nomenclatural details of a scientific name.
scientificName	The full scientific name, with authorship and date of information, if known. When forming part of an Identification, this should be the name in the lowest level taxonomic rank that can be determined. This term should not contain identification qualifications, which should instead be supplied in the IdentificationQualifier term.
kingdom	The full scientific name of the kingdom in which the taxon is classified.
taxonRank	The taxonomic rank of the most specific name in the scientificName.
decimalLatitude	The geographic latitude (in decimal degrees, using the spatial reference system given in geodeticDatum) of the geographic centre of a Location. Positive values are north of the Equator, negative values are south of it. Legal values lie between -90 and 90, inclusive.
decimalLongitude	The geographic longitude (in decimal degrees, using the spatial reference system given in geodeticDatum) of the geographic centre of a Location. Positive values are east of the Greenwich Meridian, negative values are west of it. Legal values lie between -180 and 180, inclusive.
language	A language of the resource.
waterBody	The name of the water body in which the Location occurs.
country	The name of the country or major administrative unit in which the Location occurs.
countryCode	The standard code for the country in which the Location occurs.
datasetName	The name identifying the data set from which the record was derived.
phylum	The full scientific name of the phylum or division in which the taxon is classified.
class	The full scientific name of the class in which the taxon is classified.
order	The full scientific name of the order in which the taxon is classified.
family	The full scientific name of the family in which the taxon is classified.
genus	The full scientific name of the genus in which the taxon is classified.
genericName	The genus part of the scientificName without authorship.
specificEpithet	The name of the first or species epithet of the scientificName.
continent	The name of the continent in which the Location occurs.

## Additional information

Some of the datasets compiled for this project have additional columns (Table 6).

## Figures and Tables

**Table 1.** Data collection from Venezuela used in the compilation, including number of records and references from OBIS/GBIF.

**Partial dataset**	**Dataset title**	**No. records**	**Resource type**	**Resource citation**
1	Benthic macroalgae from the coasts of Venezuela 1822-2021	10,438	Occurrence	Gómez Acevedo and Carballo Barrera (2022)
2	Zooplankton of Morrocoy National Park 2000-2002	8,066	Sampling event	Zoppi de Roa et al. (2022)
3	Diversidad de Foraminiferos en Venezuela	4,506	Occurrence	Carvajal-Chitty (2022)
4	Records of the vertical distribution of planktonic copepods in the Southern Caribbean	2,268	Sampling event	Cervigón and Scott-Frías (2022)
5	Spatial and temporal characterisation of zooplankton in Los Roques Archipelago (Venezuela)	1,824	Sampling event	Casanova et al. (2022)
6	Esponjas asociadas a raíces de *Rhizophoramangle* del Parque Nacional Morrocoy	1,414	Sampling event	Pérez (2022)
7	IOV - Distribución espacial y temporal del fitoplancton en el Golfo de Cariaco	1,144	Sampling event	Calvo-Trujillo et al. (2022)
8	Biodiversidad Marina del Parque Nacional Laguna de La Restinga	1,019	Occurrence	Lira (2022e)
9	Marine Fishes from Archipielago los Roques, Venezuela	861	Occurrence	Rodriguez et al. (2022)
10	Megabenthos biodiversity of the northwest coast of Paraguana Peninsula (Venezuela)	807	Sampling event	Lopez (2022)
11	IOV Fitoplancton del Saco del Golfo de Cariaco, Venezuela	739	Sampling event	Márquez-Rojas et al. (2022a)
12	Biodiversidad de Moluscos de la isla de Margarita	647	Occurrence	Lira (2022f)
13	Macroalgas del Parque Nacional Laguna de La Restinga, isla de Margarita, Venezuela	613	Sampling event	Lira (2022d)
14	Peces asociados a los arrecifes Coralinos del Parque Nacional Morrocoy, Venezuela	608	Sampling event	Rodriguez-Quintal et al. (2022b)
15	Peces de las lagunas costeras de Isla Margarita, Venezuela	475	Sampling event	Lira (2022c)
16	IOV Copépodos del Golfo de Cariaco, Venezuela	447	Sampling event	Marquez et al. (2022a)
17	Poliquetos criptobentonicos en *Orbicellaannularis* en arrecifes coralinos del PNA de Los Roques Venezuela	386	Sampling event	Rodriguez Fernandez et al. (2022)
18	Poliquetos fondos arenosos de Arrecifes de Coral en el PN Archipiélago de Los Roques, Venezuela	382	Sampling event	Diaz-Diaz et al. (2022)
19	IOV-UDO-ZOOPLANCTON-PLAYA COLORADA	371	Sampling event	Marquez et al. (2022b)
20	Peces asociados a raíces de manglar en el PN Morrocoy, Venezuela	279	Sampling event	López (2022)
21	Crustaceos decapodos asociados a *Stichodactylahelianthus* Isla Larga, Carabobo, Venezuela	263	Sampling event	Mariño et al. (2022)
22	Algunos Copépodos de la Fachada Atlántica de Venezuela	255	Sampling event	Camisotti and Pérez (2022)
23	Corales escleractínidos de La Orchila, Venezuela, 1976	207	Sampling event	Pérez and Urich (2022)
24	Demospongias de la Laguna La Restinga, Venezuela	199	Sampling event	Lira (2022a)
25	Componentes biológicos estudiados en el área de influencia del tramo D, poliducto SUFAZ. La primera fase del Plan de Monitoreo y Seguimiento en el marco del proyecto SUFAZ	197	Occurrence	Peralta Brichtova (2022)
26	Peces asociados a una pradera de fanerógamas marinas en el PN Archipiélago Los Roques	196	Sampling event	López et al. (2022)
27	Macroalgas del área de influencia del terminal marino de la empresa Salinera Sacosal, Araya, Estado Sucre, Venezuela	179	Sampling event	Barrios-Montilla (2022a)
28	Diversity, abundance and other ecological features of littoral brachyuran crabs from Falcon State, Venezuela	168	Sampling event	Carmona-Suarez (2022b)
29	Marine Invasive Species of Venezuela	158	Occurrence	Gonzalez (2022)
30	Peces de arrecifes coralinos de Isla Larga y Alcatraz, PN San Esteban, Venezuela	154	Sampling event	Rodríguez-Quintal et al. (2022)
31	Distribución espacial y abundancia de la Familia Corycaeidae Dana, 1852 (Copepoda: Cyclopoida) en el Golfo de Cariaco, Venezuela	149	Sampling event	Márquez-Rojas et al. (2022b)
32	Abundancia y distribución de los Branchiopoda (cladóceros) marinos del Parque Nacional Mochima, estado Sucre, Venezuela	141	Sampling event	Bravo and Marquez-Rojas (2022)
33	Community features of Swimming crabs (Portunidae) from Golfete de Cuare- Falcón- Venezuela	124	Sampling event	Carmona-Suarez (2022c)
34	Cnidarios y Poriferos del Parque Nacional San Esteban, Venezuela	117	Sampling event	Rodriguez-Quintal et al. (2022b)
35	Biodiversity of CrustaceaDecapoda from La Blanquilla Island- Venezuela	96	Sampling event	Carmona-Suarez (2022d)
36	Peces Criptobentonicos en los arrecifes coralinos del PN Archipiélago de Los Roques Venezuela	89	Sampling event	Rodriguez-Quintal et al. (2022a)
37	Composición y abundancia del plancton de la costa noreste de la bahía El Tablazo	89	Sampling event	Guerrero-Rios and Hernandez (2022a)
38	Moluscos Arrecifes Coralinos San Esteban Carabobo Venezuela	88	Sampling event	Alvarez-Barco et al. (2022)
39	Abundancia y Distribución de *Temoraturbinata* y *Temorastylifera* en el Parque Nacional Mochima, Venezuela	81	Sampling event	Colina-Romero and Marquez-Rojas (2022)
40	Macroalgas de la bahía de Macuro, estado Sucre, Venezuela. Estudio de línea base previo a la mejora del muelle de Macuro	63	Sampling event	Barrios-Montilla (2022b)
41	Cnidarios Arrecifes Coralinos de Playa Mero, Cayo Sombrero y Peraza PN Morrocoy, Venezuela	59	Sampling event	Rodriguez-Quintal et al. (2022a)
42	Diversity and geographic distribution of brachyuran crabs from the Callapidae family in Venezuela	55	Sampling event	Carmona-Suarez (2022e)
43	Crustaceos decapodos de islotes Caribe y Los Lobos, Venezuela	50	Sampling event	Lira (2022b)
44	Swimming crabs, Portunidae, from La Vela de Coro- Falcon-Venezuela	42	Sampling event	Carmona-Suarez (2022h)
45	Peces presentes en praderas de fanerogamas en Boca Seca (PN Morrocoy) y Laguna de Yapascua (PN San Esteban) Venezuela	39	Sampling event	Rodriguez Fernandez et al. (2022)
46	Records of cephalopod paralarvae (Mollusca: Cephalopoda) in the Caribbean and Venezuelan Atlantic Ocean	38	Sampling event	Stella Chacin and Scott-Frías (2022)
47	Diversity of littoral peneid shrimps in Falcon State, Venezuela	37	Sampling event	Carmona-Suarez (2022f)
48	Composition and abundance of decapod crustaceans in mixed seagrass meadows in the Paraguaná Peninsula, Venezuela	34	Sampling event	Carmona-Suarez (2022g)
49	Geographical distribution and abundance of the land blue crab *Cardisomaguanhumi* (Brachyura, Gecarcinidae) in Venezuela	34	Sampling event	Carmona-Suarez (2022a)
50	Diversidad de Corales y especies asociadas en el ecosistema coralino de Adícora, Península de Paraguaná, Venezuela	33	Sampling event	Gomez (2022)
51	Ictioplancton en cinco puntos del sector San Carlos del Lago de Maracaibo: composición distribución y abundancia	32	Sampling event	Guerrero-Rios and Hernandez (2022b)
52	Poliquetos holoplanctónicos (Annelida: Polychaeta) de la plataforma norte de la península de Paria y golfo de Paria, Venezuela	26	Sampling event	Cardenas-Oliva et al. (2022)
53	Macroinvertebrados bentónicos en un transecto ubicado entre Punta Espada y Punta Macolla, Golfo de Venezuela	20	Sampling event	Hernández (2022a)
54	Macroinvertebrados bentónicos del muro de San Carlos- Zulia- Venezuela	20	Sampling event	Hernández (2022b)
55	Population features of *Cardisomaguanhumi* in Nueva Carenero- Miranda State- Venezuela	16	Sampling event	Carmona-Suarez (2022i)
56	Spatial distribution and population features of the decorator crab *Omalacanthabicornuta* (former Microphrysbicornutus) in Buchuaco- Falcon State- Venezuela	15	Sampling event	Carmona-Suarez (2022j)
57	Diversity and ecological features of Majidae crabs from Morrocoy National Park - Venezuela	12	Sampling event	Carmona-Suarez (2022l)
58	Diversity, abundance and ecological features of swimming crabs (Brachyura; Portunidae) from Boca de Hueque, Venezuela	8	Sampling event	Carmona-Suarez (2022k)
59	A new species of the Stenopodidean shrimp genus *Spongicola*, representing the first record of the genus from the Atlantic Ocean	4	Sampling event	Rodriguez-Quintal and Goy (2022)

**Table 2.** Sampling information source.

**Data source**	**Number of records**	%
Scientific journal	21,754	53.21
Grey literature *	16,243	39.73
Books	1,863	4.56
Catalogues	1,021	2.50

* Technical reports, Project reports and Thesis.

**Table 3.** Occurrences recorded within Marine Protected Areas (MPA).

**MPA**	**No. of records**
Morrocoy National Park	11,548
Archipiélago Los Roques National Park	6,389
Laguna de la Restinga National Park	2,587
Cuare Wildlife Refuge	2,393
Mochima National Park	1,605
San Esteban National Park	988
Península de Paria National Park	188
Médanos de Coro National Park	103
Ciénaga Los Olivitos National Park	71
Laguna Boca de Caño Wildlife Refuge	50
Laguna de Tacarigua National Park	13
Isla de Aves Wildlife Refuge	3
Hueque-Sauca Wildlife Reserve	2

**Table 4.** Number of records by Phylum represented in this collection.

**Phylum**	**No. Records**	**Proportion of total records (%)**
Arthropoda	10,642	26.03
Rhodophyta	6,306	15.43
Chordata	4,657	11.39
Foraminifera	4,619	11.30
Chlorophyta	3,163	7.74
Ochrophyta	2,478	6.06
Mollusca	1,879	4.60
Porifera	1,743	4.26
Cnidaria	1,264	3.09
Annelida	1,212	2.96
Myxozoa	987	2.41
Chaetognatha	789	1.93
Echinodermata	239	0.58
Haptophyta	236	0.58
Platyhelminthes	125	0.31
Hemichordata	87	0.21
Ciliophora	77	0.19
Bryozoa	64	0.16
Euglenozoa	56	0.14
Cyanobacteria	54	0.13
Nemertea	46	0.11
Phoronida	35	0.09
Nematoda	19	0.05
Rotifera	10	0.02
Ctenophora	7	0.02
Charophyta	5	0.01
Tracheophyta	5	0.01
Basidiomycota	2	< 0.01
Brachiopoda	1	< 0.01
Cryptophyta	1	< 0.01

**Table 5.** Considering the IUCN Red List categories.

**IUCN**	**Species**	**No. Records**	**Phylum and family**
Critically Endangered (CR)	*Acroporacervicornis*	11	Cnidaria, Acroporidae
	*Acroporapalmata*	11	Cnidaria, Acroporidae
	*Sphyrnalewini*	4	Chordata, Sphyrnidae
	*Sphyrnamokarran*	3	Chordata, Sphyrnidae
	*Epinephelusstriatus*	2	Chordata, Serranidae
Endangered (EN)	*Orbicellaannularis*	22	Cnidaria, Merulinidae
	*Orbicellafaveolata*	11	Cnidaria, Merulinidae
	*Pseudobatospercellens*	4	Chordata, Rhinobatidae
	*Carcharhinusperezii*	3	Chordata, Carcharhinidae
	*Carcharhinussignatus*	3	Chordata, Carcharhinidae
	*Carcharhinusplumbeus*	2	Chordata, Carcharhinidae
	*Isurusoxyrinchus*	2	Chordata, Lamnidae
	*Aetobatusnarinari*	1	Chordata, Myliobatidae
	*Carcharhinusobscurus*	1	Chordata, Carcharhinidae
Vulnerable (VU)	*Hippocampuserectus*	10	Chordata, Syngnathidae
	*Pomatomussaltatrix*	9	Chordata, Pomatomidae
	*Agaricialamarcki*	5	Cnidaria, Agariciidae
	*Coryphopteruspersonatus*	5	Chordata, Gobiidae
	*Carcharhinusfalciformis*	4	Chordata, Carcharhinidae
	*Dichocoeniastokesii*	4	Cnidaria, Meandrinidae
	*Carcharhinuslimbatus*	4	Chordata, Carcharhinidae
	*Ginglymostomacirratum*	4	Chordata, Ginglymostomatidae
	*Lachnolaimusmaximus*	4	Chordata, Labridae
	*Carcharhinussignatus*	3	Chordata, Carcharhinidae
	*Coryphopteruslipernes*	3	Chordata, Gobiidae
	*Negaprionbrevirostris*	3	Chordata, Carcharhinidae
	*Orbicellafranksi*	3	Cnidaria, Merulinidae
	*Epinephelusmorio*	2	Chordata, Serranidae
	*Lutjanuscyanopterus*	2	Chordata, Lutjanidae
	*Mycetophylliaferox*	2	Cnidaria, Faviidae
	*Mycteropercainterstitialis*	2	Chordata, Serranidae
	*Carcharhinusplumbeus*	2	Chordata, Carcharhinidae
	*Epinephelusmorio*	2	Chordata, Serranidae
	*Lutjanuscyanopterus*	2	Chordata, Lutjanidae
	*Mycetophylliaferox*	2	Cnidaria, Faviidae
	*Mycteropercainterstitialis*	2	Chordata, Serranidae
	*Alopiassuperciliosus*	1	Chordata, Alopiidae
	*Carcharhinusleucas*	1	Chordata, Carcharhinidae
	*Coryphopterustortugae*	1	Chordata, Gobiidae
	*Cynoscionacoupa*	1	Chordata, Sciaenidae
	*Dendrogyracylindrus*	1	Cnidaria, Meandrinidae
	*Epinephelusitajara*	1	Chordata, Serranidae
	*Rhizoprionodonlalandii*	1	Chordata, Carcharhinidae
	*Rhomboplitesaurorubens*	1	Chordata, Lutjanidae
	*Alopiassuperciliosus*	1	Chordata, Alopiidae
	*Coryphopterustortugae*	1	Chordata, Gobiidae
	*Dendrogyracylindrus*	1	Cnidaria, Meandrinidae
	*Epinephelusitajara*	1	Chordata, Serranidae
	*Megalopsatlanticus*	1	Chordata, Megalopidae
	*Rhizoprionodonlalandii*	1	Chordata, Carcharhinidae
	*Rhomboplitesaurorubens*	1	Chordata, Lutjanidae
Near Threatened (NT)	*Lutjanussynagris*	18	Chordata, Lutjanidae
	*Lupinoblenniusvinctus*	15	Chordata, Blenniidae
	*Poritesbranneri*	14	Cnidaria, Poritidae
	*Lutjanusanalis*	13	Chordata, Lutjanidae
	*Scarusguacamaia*	11	Chordata, Scaridae
	*Albulavulpes*	10	Chordata, Albulidae
	*Narcinebrasiliensis*	8	Chordata, Narcinidae
	*Mycteropercabonaci*	6	Chordata, Serranidae
	*Balistesvetula*	4	Chordata, Balistidae
	*Galeocerdocuvier*	4	Chordata, Carcharhinidae
	*Hypanusamericanus*	4	Chordata, Dasyatidae
	*Agariciatenuifolia*	3	Cnidaria, Agariciidae
	*Carcharhinusaltimus*	2	Chordata, Carcharhinidae
	*Gymnuramicrura*	2	Chordata, Gymnuridae
	*Mustelusnorrisi*	2	Chordata, Triakidae
	*Hexanchusgriseus*	1	Chordata, Hexanchidae
	*Hexanchusnakamurai*	1	Chordata, Hexanchidae
	*Hypanusguttatus*	1	Chordata, Dasyatidae
	*Musteluscanis*	1	Chordata, Triakidae
	*Mycteropercavenenosa*	1	Chordata, Serranidae
	*Prionaceglauca*	1	Chordata, Carcharhinidae

**Table 6.** Additional columns present in some of the datasets compiled.

**Column label**	**Column description**
scientificNameAuthorship	The authorship information for the scientificName formatted according to the conventions of the applicable nomenclaturalCode.
institutionCode	The name (or acronym) in use by the institution having custody of the object(s) or information referred to in the record.
collectionCode	The name, acronym, coden or initialism identifying the collection or dataset from which the record was derived.
catalogNumber	An identifier (preferably unique) for the record within the dataset or collection.
recordedBy	A list (concatenated and separated) of names of people, groups or organisations responsible for recording the original Occurrence. The primary collector or observer, especially one who applies a personal identifier (recordNumber), should be listed first.
individualCount	The number of individuals present at the time of the Occurrence.
lifeStage	The age class or life stage of the Organism(s) at the time the Occurrence was recorded.
preparations	A preparation or preservation method for a specimen.
disposition	The current state of a specimen with respect to the collection identified in collectionCode or collectionID.
associatedReferences	A list (concatenated and separated) of identifiers (publication, bibliographic reference, global unique identifier, URI) of literature associated with the Occurrence.
associatedTaxa	A list (concatenated and separated) of identifiers or names of taxa and the associations of this Occurrence to each of them.
occurrenceRemarks	Comments or notes about the Occurrence.
organismRemarks	Comments or notes about the Organism instance.
eventID	An identifier for the set of information associated with an Event (something that occurs at a place and time). May be a global unique identifier or an identifier specific to the data set.
parentEventID	An identifier for the broader Event that groups this and potentially other Events.
eventTime	The time or interval during which an Event occurred.
month	The integer month in which the Event occurred.
day	The integer day of the month on which the Event occurred.
verbatimEventDate	The verbatim original representation of the date and time information for an Event.
habitat	A category or description of the habitat in which the Event occurred.
samplingProtocol	The names of, references to, or descriptions of the methods or protocols used during an Event.
sampleSizeValue	A numeric value for a measurement of the size (time duration, length, area or volume) of a sample in a sampling event.
sampleSizeUnit	The unit of measurement of the size (time duration, length, area or volume) of a sample in a sampling event.
samplingEffort	The amount of effort expended during an Event.
eventRemarks	Comments or notes about the Event.
locationID	An identifier for the set of location information (data associated with dcterms:Location). May be a global unique identifier or an identifier specific to the dataset.
island	The name of the island on or near which the Location occurs.
stateProvince	The name of the next smaller administrative region than country in which the Location occurs.
locality	The specific description of the place.
locationAccordingTo	Information about the source of this Location information.
locationRemarks	Comments or notes about the Location.
coordinateUncertaintyInMetres	The horizontal distance (in metres) from the given decimalLatitude and decimalLongitude describing the smallest circle containing the whole of the Location.
coordinatePrecision	A decimal representation of the precision of the coordinates given in the decimalLatitude and decimalLongitude.
footprintWKT	A Well-Known Text (WKT) representation of the shape (footprint, geometry) that defines the Location.
georeferencedBy	A person, group or organisation who determined the georeference (spatial representation) for the Location.
georeferencedDate	The date on which the Location was georeferenced.
georeferenceProtocol	A description or reference to the methods used to determine the spatial footprint, coordinates and uncertainties.
georeferenceSources	A map, gazetteer or other resource used to georeference the Location.
georeferenceRemarks	Notes or comments about the spatial description determination, explaining assumptions made in addition or opposition to the those formalised in the method referred to in georeferenceProtocol.
identificationQualifier	A brief phrase or a standard term to express the determiner's doubts about the Identification.
typeStatus	A nomenclatural type (type status, typified scientific name, publication) applied to the subject.
identifiedBy	A list of names of people, groups or organisations who assigned the Taxon to the subject.
identificationReferences	A list of references used in the Identification.
identificationRemarks	Comments or notes about the Identification.
acceptedNameUsage	The full name, with authorship and date information if known, of the currently valid (zoological) or accepted (botanical) taxon.
infraspecificEpithet	The name of the lowest or terminal infraspecific epithet of the scientificName, excluding any rank designation.
verbatimTaxonRank	The taxonomic rank of the most specific name in the scientificName as it appears in the original record.
verbatimIdentification	A string representing the taxonomic identification as it appeared in the original record.
minimumDepthInMetres	The lesser depth of a range of depth below the local surface, in metres.
maximumDepthInMetres	The greater depth of a range of depth below the local surface, in metres.
